# Multicentric reticulohistiocytosis: a case report

**DOI:** 10.1186/s13104-018-3753-3

**Published:** 2018-09-04

**Authors:** Azadèh Farokhi, Richard M. van Vugt, Rick Hoekzema, Michael T. Nurmohamed

**Affiliations:** 1 VU University Medical Center, De Boelelaan 1117, 1081 HV Amsterdam, The Netherlands; 2 Rheumatology Department, VUMC Amsterdam, De Boelelaan 1117, 1081 HV Amsterdam, The Netherlands; 3 Dermatology Department, VUMC Amsterdam, De Boelelaan 1117, 1081 HV Amsterdam, The Netherlands

**Keywords:** Multicentric reticulohistiocytosis, Non-langerhans cell histiocytosis, Histiocytosis

## Abstract

**Background:**

Multicentric reticulohistiocytosis is a rare form of non-langerhans cell histiocytosis presenting with skin changes and erosive arthritis. Infiltration of histiocytes and multinucleated giant cells are typical histological findings and confirm the diagnosis.

**Case presentation:**

This case report describes a newly diagnosed case of multicentric reticulohistiocytosis in a healthy 26-year-old female originally from the Philippines. Eruption of papules and nodules on the hands and pain in multiple joints were the main complaints at the initial presentation. Radiographical findings of erosions in the small hand and feet joints were impressive. Initial histological findings did not match the clinical image, although later the clinical diagnosis was supported by histological findings in additional biopsies.

**Conclusions:**

Although initial histological findings did not match the clinical image, additional biopsies were valuable to confirm the diagnosis.

## Background

Multicentric reticulohistiocytosis (MRH) is a form of non-langerhans cell histiocytosis which is rarely diagnosed. The most common presentation includes skin changes, mostly papulo-nodular eruptions in the upper extremities, and erosive arthritis. Internal organs are sometimes affected as well, which is evident from reports of pleural and pericardial effusion in patients with this disease [1]. Goltz and Laymon proposed the name multicentric reticulohistiocytosis in 1954 because of the multifocal origin and systemic nature of the disease [2]. Histologically, an infiltration of histiocytes and multinucleated giant cells can be found in lesions [3]. The prevalence of multicentric reticulohistiocytosis is not exactly known, approximately 300 cases have been reported in medical literature. A significant number of cases were associated with malignant disease, such as breast cancer, melanoma, lung cancer and cancer of colonic origin [1, 3]. The treatment of multicentric reticulohistiocytosis is not well protocolled and mostly empirical due to the unknown etiology [4]. The course and outcome of multicentric reticulohistiocytosis are unpredictable and although the disease is often self-limiting, joint deformities remain as non-denying remnants of the disease [3]. Due to the destructive nature of the arthritis in about 45% of the cases, early recognition of multicentric reticulohistiocytosis is essential and awareness among clinicians desired.

## Case presentation

We present a case of a healthy 26-year-old female of Philippine origin who presented to our outpatient clinic with a progressive eruption of cutaneous papules and nodules. Her medical history reported nodules/papules on the hands 7 years before, for which she successfully received prednisone in the Philippines with only a few residual lesions. Recently, over a course of 2 months, she noticed an increasing number of cutaneous nodules and papules; starting on her hands subsequently spreading to her wrists and forearms. Pain arose from these nodules when bumped onto a surface. Simultaneously, our patient suffered from pain and stiffness in hands, feet, elbows, shoulders and knees. Over these 2 months she felt increasingly tired. Our patient also reported a red, warm rash covering her cheeks at the start of the symptoms, which had subsided by the time of investigation. There were no signs of systemic illness, inflammation or preceding infections.

Three weeks before our initial investigation she had tried prednisone 30 mg for 3 days. Since then, she reported the occurrence of new skin lesions, a swollen face and palpitations. At the time of investigation she only sporadically used NSAIDs to control the pain.

**Fig. 1:**
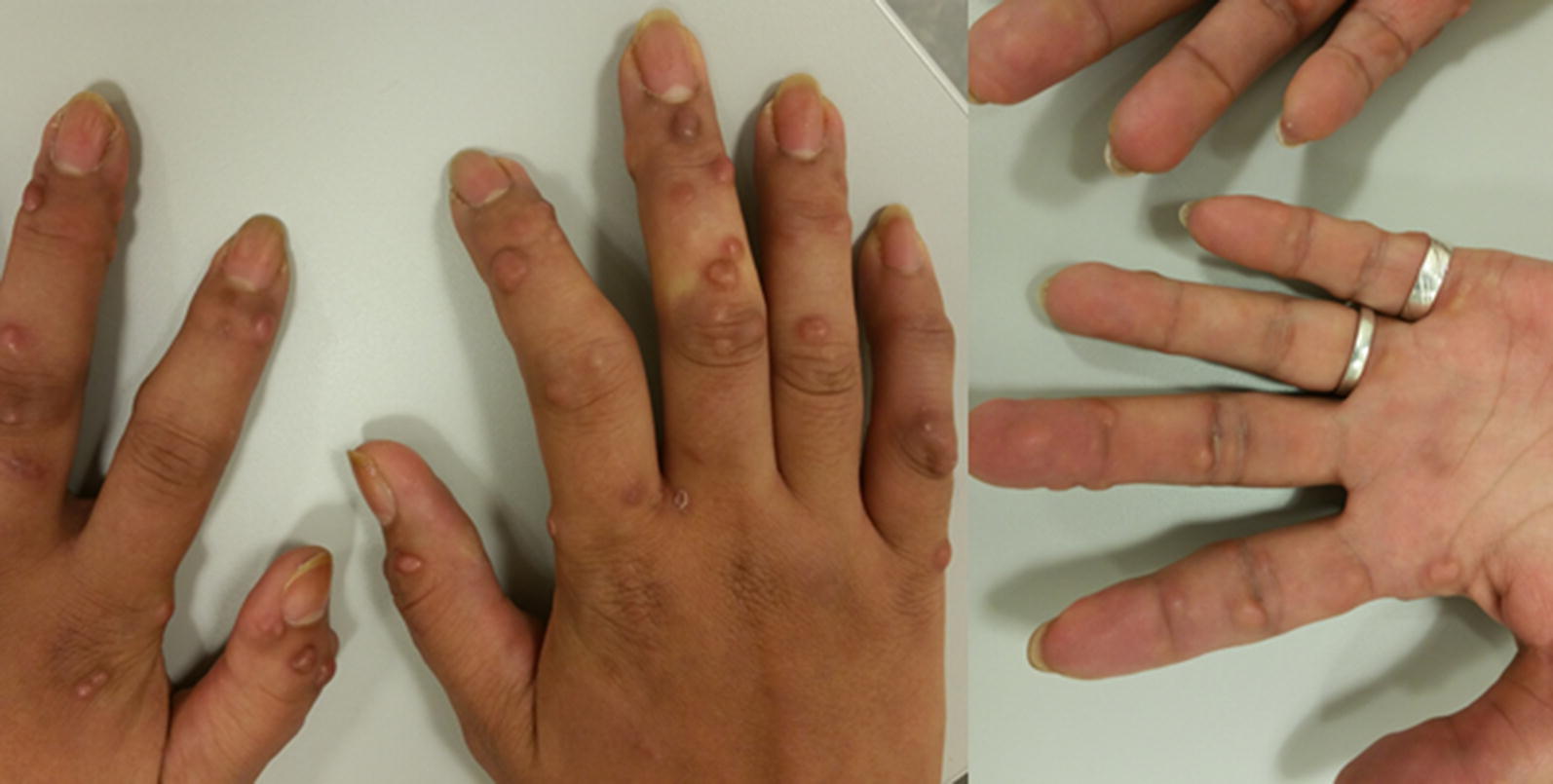
Firm nodules and papules (0.5–1.0 cm) on the hands at time of presentation

**Fig. 2:**
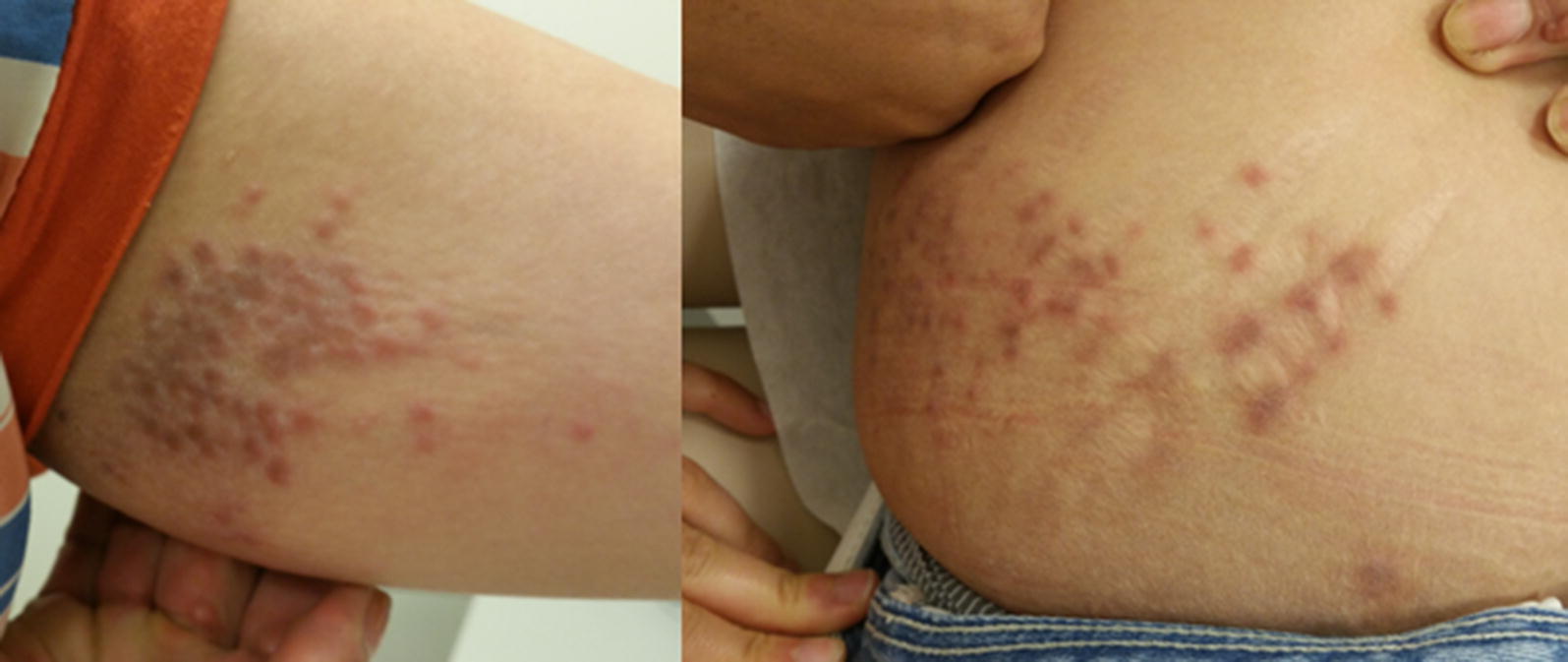
Patches of grouped, flat, red/brown papules which arose after use of prednisone, 30 mg for 3 days

On clinical examination numerous firm, either reddish-brown or skin-colored papules and nodules were seen and felt mainly on the hands and forearms, varying in size from 0.5 to 1 cm (Fig. 1). In addition, papules were found in pre-existing scars on the left shoulder and both knees. Apart from these papules and nodules, a few patches of grouped, flat, red/brown papules were seen on the inner side of the upper arms, on the hips, thighs and neck (Fig. 2).

**Fig. 3:**
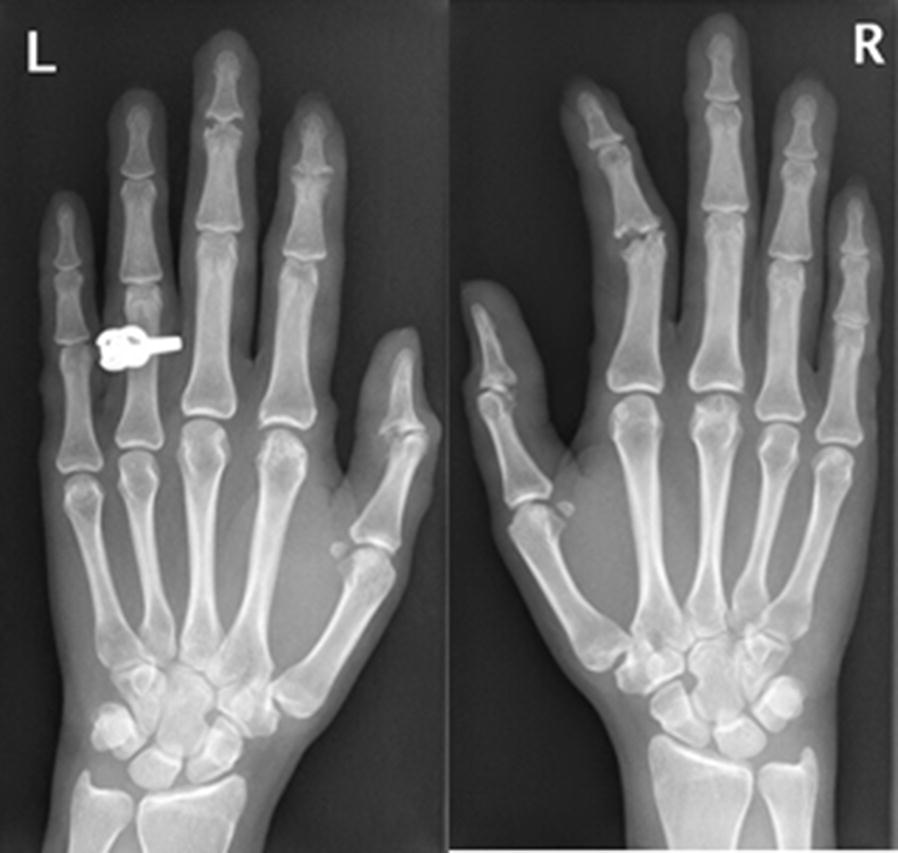
Radiographs of the hands, showing impressive erosions and destruction of small joints

At the time of examination, pain was present on palpitation of many of the small hand joints [distal interphalangeal (DIP), proximal interphalangeal (PIP) and metacarpophalangeal (MCP)], wrists, elbows and knees. Arthritis was present in several PIPs and in the wrist. Furthermore, her face appeared to be swollen. There was no lymphadenopathy. Laboratory examination revealed a normal level of ESR (erythrocyte sedimentation rate), negative antinuclear (ANA) and anti-cyclic citrullinated peptide (anti-CCP) antibodies and the IgM Rheumatoid factor was borderline positive (6.3, ref < 5.0 IU/ml). Other serum and urine laboratory tests were unremarkable. Radiographs of the hands and feet, however, showed impressive erosions and destruction of many small joints (Fig. 3) and a radiograph of the lungs showed a closed right pleural sinus.

**Fig. 4:**
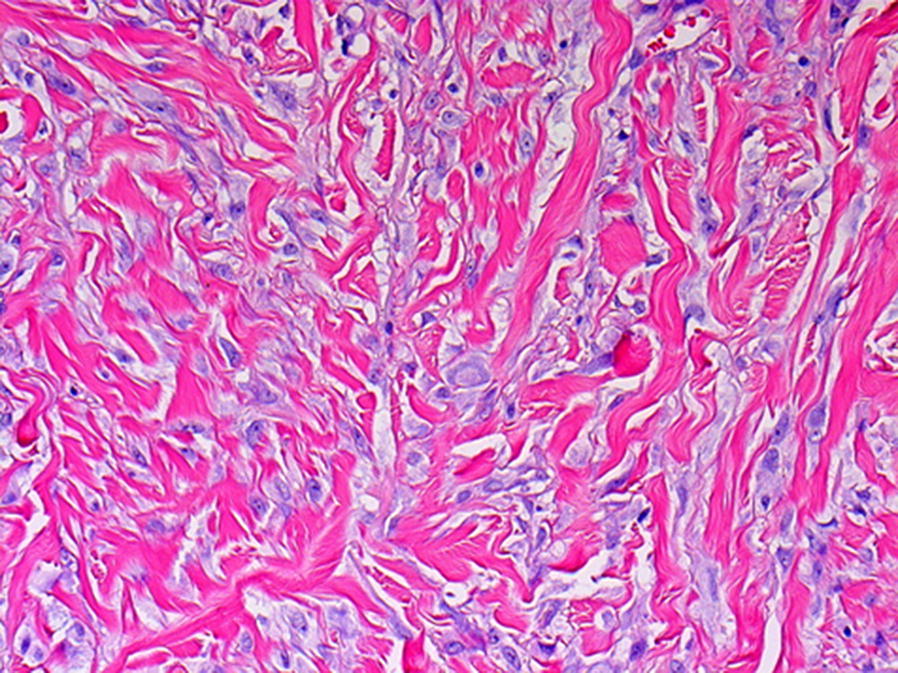
Numerous large histiocytes with sharply delineated nuclear membranes and prominent nucleoli are scattered through the dermal collagen. In the center a multinucleate giant cell is present (hematoxylin and eosin, original magnification ×200). Most of the histiocytes stained positive for CD68, whereas immunostaining for CD1a (Langerhans cells) was negative (not shown)

Punch biopsies were taken from the nodules on the hands and from the plaque on the upper arm. Initially, a histological pattern of dermal scarring/fibrosis was found, without histological features of multicentric reticulohistiocytosis. However, additional biopsies showed CD-68 positive macrophages and eosinophilic granulocytes between the collagen fibers in the dermis, in agreement with a form of non-Langerhans histiocytosis (Fig. 4).

The PET/CT scan revealed FDG-avidity in the muscles of the thighs and buttocks, the corresponding points were painful to the touch according to our patient. Also, painful joints showed slightly more uptake when compared to the representative opposite joints.

Because of the reported association with underlying malignancies [3–5], additional investigations were performed. Malignancy became very unlikely after laboratory tests, ultrasound of the abdomen, mammography, PET/CT scan and an additional MRI scan for further imaging of suspicious lesions in one of the mammae.

The initiated therapy consisted of prednisone, methotrexate and risedronate together with folic acid and calcium/vitamin D. This choice was based on an overview of different treatment regimens and outcomes published recently [1]. Prednisone was started on 30 mg and tapered to 7.5 mg in 9 weeks. Methotrexate was introduced to a final dosage of 25 mg/week over 8 weeks. Risedronate was chosen as bisphosphonates are considered to be of additional benefit in the treatment of multicentric reticulohistiocytosis [6]. Since the patient is in her reproductive age, we chose the bisphosphonate with the least implications for future pregnancy.

After 8 weeks of therapy, the joint pain had subsided and our patient was better able to move without pain. The skin lesions, however, persisted over these first 2 months of therapy. A few months later, arthritis of the right elbow was identified but the skin lesions seemed to improve and there were less complaints of itchiness.

## Discussion and conclusions

This case report was written to inform specialists about the clinical image of multicentric reticulohistiocytosis and the initial nonspecific histological findings. Since knowledge about the course of the disease and the resulting disabilities is limited, awareness is warranted among clinicians and experience should be shared in order to improve clinical care for patients with multicentric reticulohistiocytosis.

## Abbreviations

MRH: multicentric reticulohistiocytosis
